# Cutaneous Side Effects of Modern Targeted Therapy and Immunotherapy in Patients with Dermatological Malignancies

**DOI:** 10.3390/cancers15123126

**Published:** 2023-06-09

**Authors:** Kerasia-Maria Plachouri, Vaia Florou, Vasileios Georgiou, Sophia Georgiou

**Affiliations:** 1 Dermatology Department, University General Hospital of Patras, University of Patras, 265 04 Rio, Greece; georgiou@med.upatras.gr; 2 Division of Oncology, Department of Medicine, Huntsman Cancer Institute, University of Utah, Salt Lake City, UT 841112, USA; 3 School of Medicine, University General Hospital of Patras, University of Patras, 265 04 Rio, Greece

**Keywords:** immune checkpoint inhibitors, targeted therapy, cutaneous side effects, melanoma, basal cell carcinoma, squamous cell carcinoma

## Abstract

**Simple Summary:**

Cutaneous side effects are among the most frequently reported adverse reactions of modern dermato-oncological therapies, such as immune checkpoint inhibitors and targeted therapies. This study aims to provide a detailed overview of the cutaneous toxicity profile of these treatments to facilitate physicians’ early recognition of these side effects. Furthermore, we aim to accentuate the need for a dermatological evaluation of the affected patients, as it can significantly affect the patient’s quality of life and the decision to continue treatment.

**Abstract:**

The advent of immunotherapy and targeted therapies in treating dermatological malignancies has dramatically changed the landscape of dermato-oncology in recent years. Their superior efficacy compared to previous therapeutic options, such as chemotherapy, has resulted in their use in treating devastating malignancies, such as melanoma or unresectable/metastatic basal cell and squamous cell carcinoma. Skin toxicity is a critical safety consideration, among other adverse reactions, that can occur under treatment with these agents. This article aims to summarize the cutaneous side effects of immune checkpoint inhibitors and targeted dermato-oncological therapies. Although the skin side effects of these agents are primarily mild, they can occasionally affect the decision for treatment continuation and the quality of life of the affected patients. Therefore, physicians must be acquainted with the specific cutaneous toxicity profile of such treatments to mitigate their impact on the patients and optimize the overall outcome of dermato-oncological therapy.

## 1. Introduction

Immune checkpoint-inhibitors (ICIs) and targeted therapies, such as BRAF-inhibitors (BRAFIs) or MEK-inhibitors (MEKis), have revolutionized the management of patients with melanocytic dermatological malignancies, such as melanoma [1,2]. Furthermore, significant progress has been achieved in the treatment of unresectable Nonmelanoma Skin Cancer (NMSC), such as advanced basal cell carcinomas and cutaneous squamous cell carcinomas, with the introduction of both Hedgehog inhibitors and ICIs, respectively, in the armamentarium of dermato-oncologists [3,4].

ICIs are antibodies targeting checkpoint proteins, such as T lymphocyte-associated antigen-4 (CTLA-4), programmed cell death protein 1 (PD-1), or programmed death ligand 1 (PD-L1) [2,5]. These proteins are crucial for maintaining the immunological equilibrium: CTLA-4 regulates the early T-cell-associated immunologic activation, while PD-1 and PD-L1 help to regulate the late T-cell-activation in the peripheral tissues [5,6]. Furthermore, checkpoint proteins attenuate the amplitude of the immune activation and therefore allow tolerance towards the circulation of cancer cells [2,5]. Thus, the immunologic activation via immune checkpoint inhibition can result in enhanced anti-tumor activity [5,7]. Nonetheless, this mechanism of ICIs that produces such remarkable therapeutic results can also cause immune-mediated adverse reactions that often involve the skin [8].

When it comes to targeted therapies, the combination of BRAFis and MEKis has also significantly prolonged the overall survival of melanoma patients via regulation of the mitogen-activated protein kinase (MAPK) pathway [9,10]. As this pathway plays a significant role in regulating cell proliferation and differentiation, mutations in the involved genes are crucial in the development of melanoma [11]. Activating BRAF mutations are present in approximately 40–60% of patients with cutaneous melanomas, making the latter eligible for treatment with agents such as vemurafenib or dabrafenib [10]. The introduction of BRAFis was followed by the approval of MEKis, also potent inhibitors of the MAPK signaling pathway [10,11]. The combined BRAFi/MEKi therapy has been established as a first-line melanoma treatment since then, and it has demonstrated excellent therapeutic outcomes and relatively good tolerability; however, side effects have also been frequently documented, primarily due to reactivation of the MAPK signaling pathway [12,13]. It is now well established that adding a MEK inhibitor to a BRAF inhibitor has superior results in terms of survival compared to BRAF monotherapy [14]. Interestingly, the combined BRAFi/MEKi therapy seems to be less associated with skin toxicity than BRAF monotherapy [15]. Nevertheless, cutaneous adverse reactions with these agents are relatively common; therefore, physicians should be well informed concerning their management [12,16].

Finally, vismodegib and sonidegib are effective smoothened-homologue (SMO) inhibitors that have shown excellent efficacy in the treatment of advanced, non-resectable basal cell carcinomas (BCC) [3]. BCCs are characterized by an over-activated hedgehog signaling pathway due to mutations in the patched homologue 1 (PTCH1) [3]. These mutations result in uncontrolled SMO activation, thus leading to increased cell proliferation and survival [3]. Hedgehog inhibitors block this over-activated signaling pathway and prevent tumor progression [3]. However, this type of treatment is often associated with adverse events that may lead to drug discontinuation [17]. In addition, mucocutaneous side effects of Hedgehog inhibitors are very common, and their correct management is crucial for better patient tolerance [17].

This review aims to provide a summarized overview of the skin toxicities caused by ICIs and targeted tumor therapies to assist physicians in their prompt recognition. The detailed management of such manifestations is beyond the scope of this review; therefore, it will not be further elaborated.

## 2. Materials and Methods

**Figure 1:**
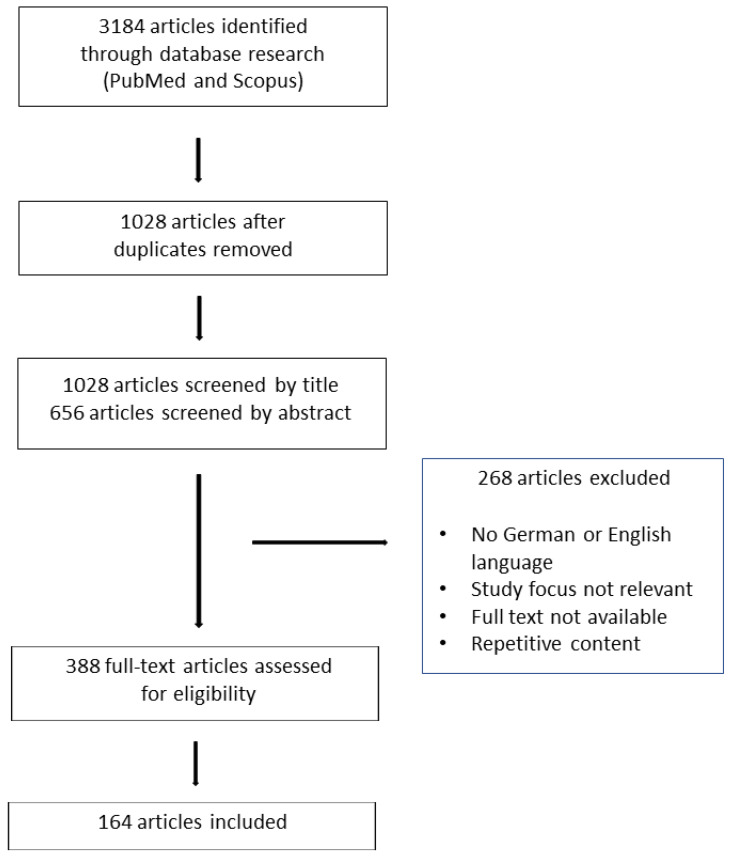
Review Flowchart.

The present study is a narrative review. Literature research was conducted by querying the following databases: MEDLINE (PubMed) and SCOPUS. The Mesh key terms that were used included the following: “ICIs” or “BRAF-inhibitor”, or “MEK-inhibitor” or” ipilimumab” or “pembrolizumab” or “nivolumab” or “relatlimab” or “cemiplimab” or “vemurafenib” or “dabrafenib” or “bimetinib” or “encorafenib” or” cobimetinib” or “trametinib” or “vismodegib” or “sonidegib” AND “skin toxicity” or “cutaneous adverse reactions” or “cutaneous side effects”. The research included articles published in English and German during the period 2010–2023. The reference lists of the papers retrieved during this process were also scanned for eligibility. Our search includes only articles that were published in the English and German languages. The selection process was performed in three steps: firstly, scanning of article titles and abstracts; secondly, exclusion of the non-relevant literature; and finally, evaluation of the remaining full-text papers (Figure 1).

## 3. Results

### 3.1. ICIs

#### 3.1.1. Maculopapular Rash

**Figure 2:**
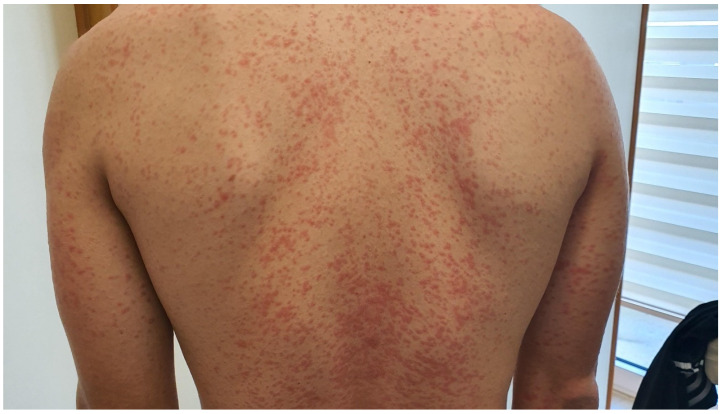
Maculopapular rash associated with Pembrolizumab in a patient with melanoma.

The maculopapular rash is the most frequently reported cutaneous irAE (immune-related adverse event) of PD-1/PD-L1 and CTLA-4 inhibitors [2]. It is most commonly seen with CTLA-4 inhibitors or under dual anti-PD1/PD-L1 and anti-CTLA therapy [18,19,20]. Although its severity can vary, grade ≥3 skin rashes, which are defined by the involvement of more than 30% of BSA (body surface area), are not frequent (Table 1) [2,5]. The rash appears as a nonspecific morbilliform or maculopapular exanthem that mainly involves the trunk, with a tendency to affect the extremities but sparing the face [21,22] (Figure 2). A prevalence of photo-exposed body areas has been documented, although that is not always the case [23]. The lesions can be asymptomatic or mildly pruritic [24]. Pruritus can either appear concomitantly with the skin reaction or precede the appearance of the lesions [2]. The outbreak can be documented as early as a few days after the administration of the first cycle or even in a delayed manner, appearing as late as several months after treatment initiation [5,25].

This rash’s most frequent histopathological characteristics include spongiotic dermatitis with associated superficial perivascular T-lymphocyte infiltrate, mimicking a cutaneous hypersensitivity reaction. In contrast, a lichenoid reaction can be documented less commonly [25,27]. Regarding other diagnostic criteria, peripheral eosinophilia concomitant with the rash appearance has been reported for both PD-1/PD-L1 and CTLA-4 inhibitors [5,24].

As this nonspecific rash can also represent an early manifestation of other more specific irAEs, such as psoriasis or bullous eruptions, a dermatological evaluation is necessary for specific classification and correct diagnosis [28]. This type of irAE is quite frequent, and, as it is benign and often self-limiting, it usually does not require aggressive management [5].

#### 3.1.2. Lichenoid Rash

Lichenoid cutaneous drug reactions constitute another class of frequently encountered skin adverse events, and some experts believe they are almost as frequent as the nonspecific maculopapular rash [2,5,25]. This type of skin eruption tends to appear later than maculopapular irAEs, several months after ICI initiation [2,29].

In terms of clinical presentation, lichenoid irAEs can appear as classical lichen planus lesions, with pruritic polygonal papules exhibiting Wickham striae, or in a papulosquamous and bullous form, with a predilection for the trunk and extremities [5,30,31]. Mucosal, inverse, or even palmoplantar distribution has also been documented, although not very frequently [23,32,33]. The intensity of pruritus can vary from mild to severe, significantly affecting the patient’s quality of life and subsequent therapeutic management [2].

Diagnosis requires a histopathological examination, with which signs of interface dermatitis with a band-like lymphocytic infiltrate along the dermo-epidermal junction, vacuolization, and apoptotic keratinocytes can be documented [25,31,33]. In addition, hypergranulosis, acanthosis, spongiosis, and occasionally parakeratosis have also been reported [25,34]. Dermoscopic criteria that can further facilitate diagnosis include the presence of Wickham striae on the lesions [2].

#### 3.1.3. Psoriasis or Psoriasiform Rash

Treatment with both PD-1/PD-L1 and CTLA-4 inhibitors entails a risk for the development of psoriasis or exacerbation of a pre-existing psoriatic disease [35,36]. Neither the exact incidence nor the detailed pathogenetic mechanism of this side effect has been completely elucidated [5]. Some suggest that this is a result of reactive overexpression of proinflammatory cytokines of the Th-17 signaling pathway as a response to the inhibition of PD-1 [37]. PD-1 normally downregulates the Th17 axis; therefore, its inhibition can generate a cascade that results in an immune-mediated T-cell activation with a shift towards a cytotoxic CD4+/CD8+ immunological status [38].

**Figure 3:**
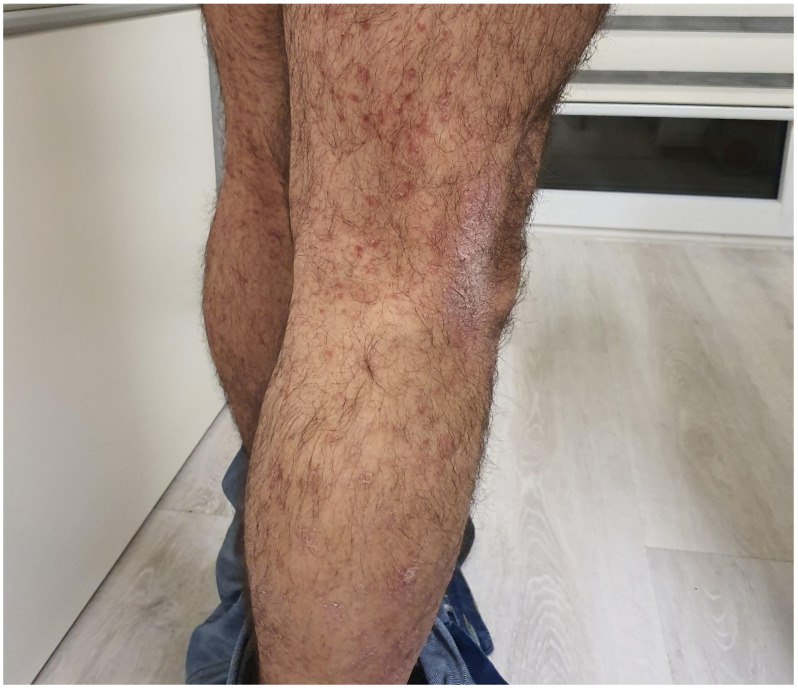
Psoriasiform lesions associated with ipilimumab in a patient with melanoma.

The clinical presentation of ICI-mediated psoriasis involves mostly the appearance of typical erythematosquamous plaques, while pustular, inverse, and guttate phenotypes have also been described [39,40] (Figure 3). Palmoplantar and scalp psoriasis, as well as the worsening of pre-existing or the appearance of newly acquired psoriatic arthritis, are also reported [2,39,40]. Histological findings do not differ from those of typical psoriasis vulgaris [41].

#### 3.1.4. Bullous Eruptions

The appearance of autoimmune bullous disorders under treatment with PD-1/PD-L1 and CTLA-4 inhibitors has been well demonstrated, with most reports indicating a higher prevalence under PD-1/PD-L1 inhibitors compared to anti-CTLA-4 inhibitors [2,42]. Prevalence is estimated to range between 1 and 8% of patients receiving ICIs, and the majority of cases are reported to occur with PD-1/PD-L1 inhibitors [43,44,45]. Exacerbation of pre-existing bullous pemphigoid has been mostly reported under anti-CTLA-4 agents [46].

Most cases of bullous irAEs involve bullous pemphigoid, with a disease onset varying from a few weeks to several months following treatment initiation [47]. Prior to the appearance of blisters, most patients notice pruritus alone or combined with erythematous and/or urticarial rashes, while mucosal involvement is rather uncommon [47,48,49]. Emerging reports on the appearance of newly acquired pemphigus vulgaris or exacerbation of pre-existing pemphigus disease under both PD-1/PD-L1 and CTLA-4 inhibitors are also present in the literature [50,51,52]. Cases of paraneoplastic pemphigus have also been reported and are associated mostly with the administration of PD-1/PD-L1 inhibitors [53,54]. Isolated cases of bullous lichenoid dermatitis, dermatitis herpetiformis, and linear IgA bullous dermatosis are also documented [2,5,27].

Diagnosis requires confirmation via histopathologic evaluation, immunohistochemistry, as well as findings from direct and indirect immunofluorescence [2]. The pathogenetic mechanism is not fully understood, but it is believed to be a result of excessive T-cell activation against bullous-pemphigoid-associated antigens that can also be expressed on the surface of tumor cells [49,55].

#### 3.1.5. Pruritus

Pruritus is among the most common cutaneous adverse reactions under ICIs [42]. The prevalence of this complication is higher under monotherapy with ipilimumab or combination therapy of ipilimumab/nivolumab, as opposed to anti-PD-L1 treatment [5,56,57,58]. It can present either alone, in combination with nonspecific maculopapular rashes, or due to other conditions such as xerosis and other ICIs-associated dermatoses, including psoriasis [20,59]. Despite its benign nature, pruritus of intense severity can significantly affect the life quality of patients, and, therefore, it should be addressed accordingly (Table 1) [2].

#### 3.1.6. Vitiligo

Vitiligo is another relatively frequent irAE, with an incidence of approximately 11% and 25% with CTLA-4 & PD-1/PDL1 inhibitors, respectively [5,14,56]. ICIs-related hypopigmentation is reported to occur as frequently as 2–8% in melanoma patients; however, its prevalence in patients with other malignities is unknown [42]. It is only sporadically mentioned as a treatment-related complication in various malignancies, including non-small cell lung cancer or soft-tissue sarcoma [60,61,62,63].

Regarding pathogenesis, a cytotoxic action of CD8+ activated T-cells against healthy melanocytes that share specific antigens with tumor melanocytes, such as Melan-A or gp100, is speculated [64,65]. The onset of this adverse reaction is usually several months after treatment initiation, and it is not a dose-related phenomenon [66].

**Figure 4:**
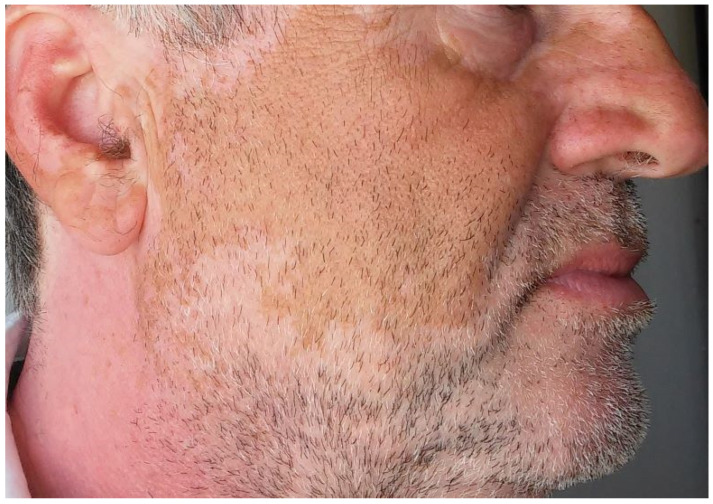
Vitiligo under pembrolizumab in a patient with melanoma.

Clinically, lesions can be either focal and/or localized or generalized with a symmetrical distribution (Figure 4 [65]. Hair depigmentation, including eyebrows and eyelashes, can occur, while reports on regression of pre-existing pigmented lesions, such as nevi and solar lentigines, are also present in the literature [23,67]. Interestingly, a case series reporting hair repigmentation in 14 patients treated with PD-1/PD-L1 inhibitors for lung cancer is also present in the literature [68,69]. Although vitiligo does not seem to resolve after treatment discontinuation, its occurrence is frequently associated with a favorable therapeutic outcome and prolonged overall survival [70,71,72].

#### 3.1.7. Hair and Nail Toxicity

**Figure 5:**
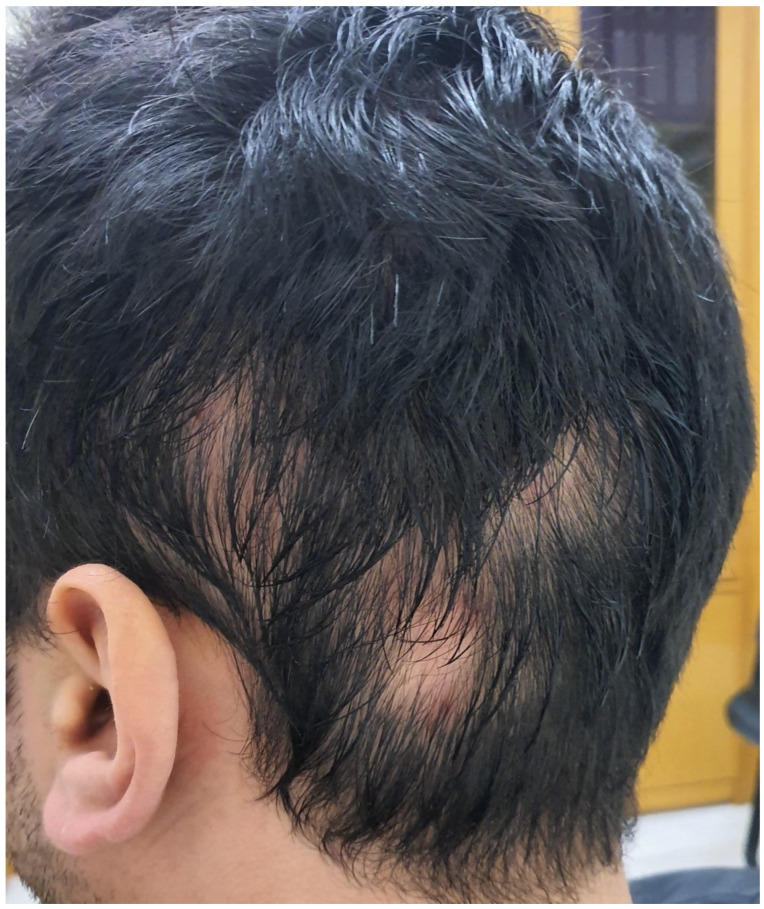
Alopecia areata under pembrolizumab in a patient with melanoma.

The most frequently encountered hair disorder among melanoma patients that receive ICIs is alopecia areata, which can be either focal or diffuse [23,73,74] (Figure 5). It tends to be more severe under CTLA-4 inhibitors than PD-1/PD-L1 inhibitors [5]. The pathogenic mechanism behind this irAE is believed to be a CD4+ and CD8+ T cell-mediated immune reaction triggered by PD-L1 that is present in cells of the hair follicle sheath [75].

The onset of ICI-related alopecia areata is usually 3–6 months after treatment initiation [73]. In the case of hair regrowth in the affected areas, the newly grown hair could show signs of poliosis or a difference in hair structure [73,76]. As mentioned above, isolated cases of hair repigmentation under PD-1/PD-L1 treatment have also been documented [69]. Regarding other hair toxicities, besides alopecia areata, cases of telogen effluvium have also been reported [42].

Regarding nail toxicity, most of the isolated reported A.E.s to describe nonspecific onychodystrophia, onychomadesis, onycholysis, and paronychia [5,75].

#### 3.1.8. Mucosal Toxicity

Inflammation of the oral mucosa manifesting as nonspecific stomatitis, periodontitis, and lichenoid reaction is relatively common and, in fact, better documented under treatment with PD-1/PD-L1 inhibitors, as opposed to CTLA-4 inhibitors [34,77,78].

Dysgeusia and xerostomia of diverse severity have also been documented, especially under anti-PD-1/PD-L1 treatment, and they are attributed to CD4/CD8-T-cell toxicity against the salivary glands [5,79]. Periodontitis is also believed to occur due to a T-cell-mediated inflammation that could be so severe as to provoke even tooth loss due to alveolar bone absorption [77].

Lichenoid reactions usually exhibit Wickham striae over an erythematous plaque or appear as papules, ulcers, and atrophic patches [42,80]. Lesions can also affect genital and perianal areas and can be either asymptomatic or mildly painful and sore [42]. ICI-associated mucosal toxicity might not be life-threatening; however, it can significantly interfere with the patients’ quality of life, and prompt management is necessary to avoid facing treatment modification dilemmas [75,81,82].

#### 3.1.9. Potentially Life-Threatening Adverse Reactions

Severe or even potentially life-threatening cutaneous drug reactions, such as Stevens–Johnson syndrome, toxic epidermal necrolysis, acute generalized exanthematous pustulosis (AGEP), and drug reaction with eosinophilia and systemic symptoms (DRESS), have been scarcely reported to occur under treatment with ICIs [81,83,84,85,86,87,88,89,90].

Both PD-1/PD-L1 inhibitors as well as CTLA-4 inhibitors can induce the appearance of these serious conditions [91,92]. Furthermore, these adverse reactions can occur even after a prolonged period of time from treatment initiation [86,87]. Finally, as nonspecific maculopapular rashes can precede the appearance of such potentially life-threatening eruptions, a careful dermatologic evaluation and, eventually, a histologic examination of a skin biopsy may be necessary to avoid devastating complications [8,93].

#### 3.1.10. Other Cutaneous Adverse Reactions

Other ICI-associated cutaneous adverse reactions reported as case series or case reports are summarized alphabetically in Table 2.

## 4. BRAF-Inhibitors

### 4.1. Photosensitivity

One of the most common side effects of BRAFis is photosensitivity, with an incidence ranging between 22.2% and 66.7% [9,118]. Most cases are related to vemurafenib compared to dabrafenib, and the mechanism of action is believed to be UVA-dependent photosensitivity [119,120].

The onset can be as early as a few days after treatment initiation, and it is mostly mild, although isolated more severe cases have also been documented [12,120,121]. Symptoms tend to intensify during the summer period, and the associated skin eruptions are more prevalent in sun-exposed body areas [120,121]. Facial or extra facial erythemas and actinic cheilitis of the lower lip are among the most frequent photosensitivity-associated documented skin adverse events [120]. Other symptoms include painful sunburns with or without blistering, with subsequent limitations for outdoor activities for the affected individuals [14].

### 4.2. NMSC (Nonmelanoma Skin Cancer) and Benign Cutaneous Neoplasias

The appearance of benign verrucous lesions is another frequently encountered adverse skin reaction in patients receiving BRAFis [122]. The role of HPV colonization in the pathogenesis of this side effect is controversial [14,122,123]. Verrucae vulgaris can appear on both sun-exposed and non-sun-exposed body areas, and they can appear as early as a few days after the initiation of BRAFi treatment; nonetheless, in the majority of cases, the onset is after four weeks of therapy [3,124]. Although they are benign, rare instances of malignant transformation to squamous cell carcinomas (SCC) have been reported [125]

Actinic keratoses (Aks) are also documented to occur frequently under BRAFi treatment, with an incidence among the studies ranging between 26 and 66.7% [118,123,124]. They occur mostly in sun-exposed areas, such as the scalp, face, and extremities, and they have a higher prevalence in photo-damaged individuals under BRAFis [14].

SCCs constitute the majority of NMSCs that can appear under therapy with BRAFis [9]. The mean onset time is estimated to be approximately seven weeks after treatment initiation, and they can appear de novo or on the ground of a pre-existing AK that has undergone malignant transformation [124]. Although the pathogenetic mechanism of this adverse reaction is not fully known, paradoxical activation of the MAPK signaling pathway in keratinocytes with pre-existing RAS mutations is speculated [12,126]. Similarly to AKs, SCCs also have a higher prevalence in sun-damaged skin, and they are mostly documented in patients over 49 years old [12,127]. Keratoacanthomas are well-differentiated SCCs and can also appear under BRAFi treatment [9].

### 4.3. Benign and Malignant Melanocytic Lesions

Therapy with BRAFis can induce changes in benign melanocytic nevi, such as changes of color and size or even involution of the lesions [128,129]. The mechanism behind nevi involution with BRAFis is believed to be the presence of the BRAF V600E mutation in the nevi melanocytes, which increases their susceptibility to treatment with the later agents [9]. In addition, eruptive melanocytic nevi with vemurafenib or encorafenib have also been described [130,131]. The explanation for this phenomenon, as well as for an increase in nevi size and pigmentation, is attributed to a BRAFi-assοciated proliferation of wild-type BRAF cells in the melanocytic lesions due to the activation of the RAS-RAF-MEK-ERK pathway, which induces increased cell proliferation and survival [130].

Lastly, the development of cutaneous melanomas de novo or arising from pre-existing nevi has also been described [128,132]. Newly developed melanomas can be either invasive or in situ, and the mean time of occurrence is estimated at approximately 4 to 12 weeks after treatment initiation [132,133].

### 4.4. Maculopapular Rash

Nonspecific maculopapular or morbilliform cutaneous eruptions are among the most frequent BRAFi-associated cutaneous adverse events [3,12]. The severity can range from grade 1 to grade 3; their incidence is documented to be between 64% and 75% in patients on BRAFi therapy, and the mean time of occurrence is approximately 1.6 weeks after treatment initiation [9,16]. Such eruptions can also occur in the face, trunk, and extremities [9]. Among the documented maculopapular or morbilliform eruptions are also keratosis-pilaris-like eruptions that are mostly asymptomatic, affecting arms and thighs. Notably, they are reported to occur more commonly under dabrafenib compared to vemurafenib [12,14].

### 4.5. Severe Cutaneous Adverse Reactions

Severe and potentially life-threatening cutaneous adverse events under treatment with BRAFis have also been documented. Such conditions include Stevens–Johnson syndrome, toxic epidermal necrolysis, DRESS, AGEP, and generalized bullous fixed eruption [10]. Most reported cases concern melanoma patients rather than other malignancies treated with BRAFis [10]. These types of adverse events are not frequent and tend to occur less commonly with dabrafenib–trametinib, encorafenib–binimetinib, and vemurafenib/cobimetinib, compared to vemurafenib monotherapy [134,135]. The mean time of onset is approximately 15.5 days for Steven’s–Johnson syndrome or toxic epidermal necrolysis and 11.4 days for DRESS, while a prior treatment with ICIs tends to increase the susceptibility to occurrence of a serious cutaneous adverse events [10].

### 4.6. Other Cutaneous Adverse Reactions

Other BRAFi-associated cutaneous adverse reactions, reported as case series or case reports, are summarized alphabetically in Table 3.

## 5. MEK-Inhibitors

### 5.1. Cutaneous Eruptions

The most frequently reported MEKi-associated cutaneous adverse event is a papulopustular eruption, with an incidence varying between 40% and 93% [147]. In terms of clinical presentation, this eruption tends to appear as pruritic papules and pustules in areas of high-sebum production, such as the scalp, face, and upper trunk [14]. In most documented cases, the severity of the papulopustular eruption is mild to moderate [148]. Such acneiform eruptions have been occasionally reported to co-exist with pruritus and erythema [148]. This type of skin toxicity is similar to the acneiform eruptions observed under epidermal growth factor receptor inhibitors (EGFRi), and it can occur as early as a few weeks under treatment initiation [14,149].

The mechanism of this adverse reaction is believed to be a MEKi-associated activation of the phosphoinositide 3-kinase (PI3K)-AKT pathway that subsequently results in an Insulin-like growing factor-1 (IGF-1)-mediated induction of sebaceous glands lipogenesis [150].

Other nonspecific cutaneous eruptions, such as maculopapular eruption with or without pruritus, have also been described under MEKis [148]. In addition, other clinical variations of maculopapular rashes have been sporadically described [151]. Patel et al. reported a case series of three patients who presented with a distinct drug hypersensitivity reaction on selumetinib, with disseminated lesions exhibiting an urticarial aspect and a characteristic central duskiness [151].

### 5.2. Other Cutaneous Findings

Other MEKi-associated dermatologic adverse reactions that are similar to EGRF-related skin toxicity include pruritus, xerosis, angular cheilitis, mucositis, paronychia, periungual fissuring, and hair disorders, such as alopecia, trichomegaly, and changes in hair texture and hair color [152]. The similarities between the skin toxicity profiles of MEKis and EGFR inhibitors are believed to derive from the direct inhibition of the MAPK pathway [153]. The safety profile of MEKis is favorable overall, causing milder cutaneous side effects compared to other systemic melanoma treatments [154].

Further MEKi-associated cutaneous adverse reactions, reported either as case series or case reports, are summarized alphabetically in Table 4.

## 6. Hedgehog-Inhibitors

### 6.1. Alopecia

Alopecia is a well-documented cutaneous side effect of vismodegib, with a prevalence as high as 63% [157]. In addition, sonidegib-associated alopecia has also been documented [158]. The pathogenetic mechanism of this adverse reaction is based on the significant role of the Hedghog pathway in early hair follicle morphogenesis; therefore, inhibitory agents could induce an arrest in normal hair follicle development [157].

The onset of alopecia is usually approximately four months after treatment initiation [157]. The hair loss is gradual, and it can also affect body hair [159]. The severity of alopecia can vary from mild to ≥50% hair loss, and its clinical presentation can be either patchy or diffuse [159]. In most cases, alopecia resolves after therapy discontinuation, and the time frame for hair regrowth is approximately 6–12 months after treatment cessation [159,160]. However, even in the case of hair regrowth, the hair density could be permanently affected [159]. Isolated reports of alopecia persisting even longer than one year after treatment cessation have also been documented; it is, therefore, important to counsel patients accordingly [157].

### 6.2. Dysgeusia

Dysgeusia is another common side effect of treatment with hedgehog inhibitors, in approximately 51–85% of patients [161]. The pathogenic mechanism of this adverse reaction is believed to be a local effect of these agents in taste buds, resulting from the inhibition of the hedgehog-pathway signaling [162]. In a study by Yang et al., taste bud size and the number of taste cells per taste bud were significantly reduced in mice under vismodegib administration [162]. It was also demonstrated that the treatment with vismodegib led to a significant reduction of cells expressing molecules such as phospholipase Cβ2 or glucagon-like peptide-1, known modulators of sweet and bitter taste sensitivity [162].

The time of onset of dysgeusia or ageusia is about 1.3 to 6.5 months after treatment initiation, and clinical symptoms can vary [159]. Some patients experience a complete loss of taste, others experience a metallic taste, while others notice an increased taste of sweet or salty sensations [159]. Occurrences of unpleasant tastes or changes in taste in alcohol have also been documented [159]. Due to this adverse reaction, a large patient group often loses interest in food intake, which can lead to severe malnutrition and weight loss [159,161].

Further cutaneous adverse reactions under treatment with hedgehog inhibitors that have been less frequently reported are summarized alphabetically in Table 5.

## 7. Conclusions and Future Directions

With the increasing use of ICIs and targeted therapies in treating dermatologic malignancies, physicians gain a better understanding of their toxicity profile, both in terms of early recognition and adequate management [10]. Although the majority of cutaneous side effects of the aforementioned treatments are relatively mild, severe adverse reactions can also occur, affecting patient satisfaction and even treatment continuation [2]. It is, therefore, evident that multidisciplinary management of these cases, involving a dermatological assessment, is critical to ensure optimal patient treatment and a successful therapeutic outcome overall.

## Figures and Tables

**Table 1:** Skin toxicity grading [26].

Adverse Reaction	Grace 1	Grade 2	Grade 3	Life-Threatening Reactions
Maculopapular rash	Macules/papules covering\10% BSA with or without symptoms (e.g., pruritus, burning, tightness)	Macules/papules covering 10–30% BSA with or without symptoms (e.g., pruritus, burning, tightness)	Macules/papules covering 30% BSA with or without associated symptoms; limiting self-care activities	-
Pruritus	Mild or localized; topical intervention indicated	Intense or widespread; intermittent; skin changes from scratching (e.g., edema, papulation, excoriations, lichenification, oozing/crusts); oral intervention indicated	Intense or widespread; constant; limiting self-care activities or sleep; oral corticosteroid or immunosuppressive therapy indicated	

**Table 2:** Less frequently reported ICI-associated cutaneous adverse reactions.

Less Frequently Encountered ICI-Associated irAEs
Acneiform rash/ papulopustular folliculitis [5,43,94]
Dermatomyositis [95,96,97]
Eruptive keratoacanthomas [98]
Erythema nodosum [99,100,101]
Grover’s disease [102,103,104]
Photosensitivity [5]
Pyoderma gangrenosum [105,106]
Sarcoidosis [107,108,109,110,111]
Sjogren’s syndrome [112]
Sweet syndrome [113,114]
Rosacea [115]
Urticaria [5]
Vasculitis [116,117]

**Table 3:** Less frequently reported BRAFi-associated cutaneous adverse events.

Less Frequently Encountered BRAFi-Associated Cutaneous Adverse Events
Acneiform eruption [123,124]
Alopecia (telogen effluvium) [136]
Basal cell carcinoma [137]
Cheilitis [9]
Granulomatous eruption [138,139,140]
Grover’s disease [141]
Hand-foot skin reaction [142]
Milia [9]
Panniculitis [143,144]
Pruritus [10,14]
Vitiligo [145,146]
Xerosis [9]

**Table 4:** Less frequently reported MEKi-associated cutaneous adverse events.

Less Frequently Encountered MEKi-Associated Cutaneous Adverse Events
Angular cheilitis [14]
Cellulitis [153,155,156]
DRESS syndrome [153,155,156]
Hair disorders [152]
Mucositis [14]
Paronychia and periungual fissuring [14]
Psoriasiform scalp dermatitis
Pruritus [153]
Teleangiectasias [14]
Urticaria [153,155,156]
Xerosis [152]

**Table 5:** Less frequently encountered cutaneous adverse reactions under treatment with hedgehog inhibitors.

Less Frequently Encountered Cutaneous Adverse Reactions under Treatment with Hedgehog Inhibitors
AGEP [163]
Cutaneous eruptions (maculopapular, papulopustular) [164]
Folliculitis [160]
Grover’s disease [164]
Hypersensitivity reactions [160]
Keratoacanthomas [165]
Stevens-Johnson syndrome/Toxic epidermal necrolysis [163]

## Abbreviations

AGEP	Acute Generalized Exanthematous Pustulosis
BRAFi	BRAF-inhibitors
BSA	Body Surface Area
CTLA-4	T lymphocyte-associated antigen-4
DRESS	Drug Reaction with Eosinophilia and Systemic Symptoms
EGFR	Epidermal Growth Factor Receptor Inhibitors
ICIs	Immune Checkpoint-Inhibitors
IGF-1	Insulin-like Growing Factor-1
irAE	Immune-related Adverse Event
MAPK	Mitogen-activated Protein Kinase
MEKi	MEK-inhibitors
NMSC	Nonmelanoma Skin Cancer
PD-1	Programmed Cell Death Protein 1
PD-L1	Programmed Death Ligand 1
PTCH1	Patched Homologue 1
SCC	Squamous Cell Carcinomas
SMO	Smoothened Homologue
